# Impact of health literacy and illness perception on medication adherence among older adults with hypertension in Iran: a cross-sectional study

**DOI:** 10.3389/fpubh.2024.1347180

**Published:** 2024-03-27

**Authors:** Towhid Babazadeh, Soheila Ranjbaran, Sara Pourrazavi, Arman Latifi, Khalil Maleki Chollou

**Affiliations:** ^1^Department of Public Health, Sarab Faculty of Medical Sciences, Sarab, Iran; ^2^Research Center of Psychiatry and Behavioral Sciences, Tabriz University of Medical Sciences, Tabriz, Iran; ^3^Department of Public Health, School of Public Health, Research Center for Evidence-Based Health Management, Maragheh University of Medical Sciences, Maragheh, Iran; ^4^Department of Nursing, Sarab Faculty of Medical Sciences, Sarab, Iran

**Keywords:** adherence, medication, hypertension, older adults, Iran

## Abstract

**Background:**

Adherence to medication is an essential factor in controlling and reducing the side effects of non-communicable diseases, particularly hypertension. Medication adherence varies in older adults due to the effects of various factors. The research aimed to examine the determinants of medication adherence among older adults with hypertension.

**Methods:**

This cross-sectional study was performed among 300 people aged 60 years or older referring to health centers in Sarab, Iran, between February and May 2023. To collect data, valid and reliable tools were applied.

**Results:**

There was a significant association between age groups, level of education, and monthly income status with adherence to medication (*p*-value <0.05). According to the results of hierarchical regression, demographic variables collectively explained 3.2% of the variance in adherence to therapeutic regimens (*p*-value = 0.143). The inclusion of illness perception at step 2, along with demographic variables, led to a further significant increase in 9.6% of the variance (*p*-value <0.001). In the final step, health literacy dimensions were added, which explained an additional 8.7% of the variance (*p*-value <0.001). In total, demographic variables, illness perception, and HL dimensions explained 21.5% of the variance in adherence to therapeutic regimens.

**Conclusion:**

According to the results, demographic variables, illness perception, and HL dimensions were the main determinants of medication adherence among older adults. Health educators should focus on creating interventions that improve medication adherence by addressing illness perception and health literacy dimensions in this particular population.

## 1 Introduction

Hypertension, also known as high blood pressure, is a chronic disease that contributes significantly to the burden of cardiovascular diseases (1). The prevalence of hypertension is increasing worldwide due to an aging population and increased exposure to lifestyle risk factors (1–3). According to the reported statistics by the Centers for Disease Control and Prevention (2015–2018), 66.7% of men aged 65–74 and 81.5% of men aged above 75, as well as 74.3% of women aged 65–74 and 86% of women aged above 75, suffer from high blood pressure (4).

Although hypertension often has no symptoms, the complications caused by it, such as stroke, cerebral infarction and cerebral hemorrhage, kidney failure, heart problems, and death, have caused this disease to be called the “silent killer” (2, 3, 5). Hypertension has some modifiable risk factors, such as smoking, inactivity, and obesity (1, 3). Reducing the risk of complications from hypertension could be achieved through drug therapy (3). Although the treatment of hypertension is a lifelong process, the continuation of treatment and its adherence can play a crucial role in controlling the disease (1) and preventing disability in people aged 65 and older (5).

Medication adherence is one of the health-related coping strategies that refers to the extent to which people’s behavior matches the health or treatment recommendations provided by healthcare providers (6). According to the literature, medication adherence can affect the therapeutic and clinical outcome, as well as health-related quality of life (3, 7, 8). Medication non-adherence is a major global health problem with significant consequences in older adults due to their chronic and multiple diseases and polypharmacy (9, 10). Patients with high blood pressure are unaware of the progress of the disease and its potential damage to various organs of the body if they do not adhere to the treatment (5, 11, 12). Demographic, medical, pharmaceutical, behavioral, and economic factors (1, 5, 13) can disrupt a person’s efforts to adherence treatment, some of which occur intentionally and some unintentionally (13). Low-income populations afflicted with chronic conditions represent a susceptible demographic when it comes to adherence to treatment, as their access to adequate pharmacological therapy may be impeded by restricted resources, thereby hindering their ability to receive the necessary ongoing medical care often necessary for the management of chronic ailments (14).

Illness perception is one of the most important factors in this regard (15). People adhere to the prescribed treatment depending on whether they correctly understand their disease or not and the relevance of the recommended treatment with the effective management of the disease (13). In other words, the way a patient perceives the illness and the benefits of prescribed medications can affect their willingness to adhere to medication regimens (15, 16). According to the literature, illness perception and positive beliefs about treatment in older adults with hypertension are associated with medication adherence (13, 15, 16).

Another important effective factor in the management of hypertension and medication adherence is health literacy (5, 17). Health literacy is the ability of an individual to obtain, interpret, understand, and translate knowledge and health information to make appropriate health decisions and improve health (18–20). It includes using the skills of reading, writing, listening, calculating, speaking, analyzing, and making decisions in a healthy situation; it is not necessarily related to years of education or general reading ability (19). Health literacy significantly affects the ability to take medication and disease knowledge in older adults (18).

Many seniors suffer from poor health literacy, with three out of four having difficulty interpreting the printed health information and more than 75% of them having difficulty with math calculations and understanding numbers, which are important for adherence to medical orders (5). Health literacy has a positive and strong relationship with medication adherence (18). Despite decades of research in medication adherence, non-adherence to treatment in patients aged over 65 years with blood pressure is still controversial (9). Considering the importance of medication adherence in older adults’ health, the present study aimed to identify the determinants of medication adherence among older patients with hypertension.

## 2 Methods

### 2.1 Participants and sampling

This cross-sectional study took place in Sarab, Iran, between February to May 2023. The participants were people aged 60 years or older referring to Sarab health centers. The sample size was calculated as 300 people based on the formula $\frac{z^{2}s^{2}}{d^{2}}$ and the information derived from a similar study (21) and a confidence level of %95, *Z* = 1.96, SD = 2.27, and mean = 5.21. The inclusion criteria for this study were individuals aged 60 years or older, diagnosed with hypertension, free from psychiatric disorders, and willing to sign a consent form to participate. The only exclusion criterion was unwillingness to take part in the study.

For sampling, a multi-state cluster random method was used. The city of Sarab comprised four health centers, each considered as a cluster. Eligible participants were randomly chosen from these centers using health records. Following an explanation of the study’s objectives, informed consent was obtained from the participants. First author, second and corresponding author were conducted face-to-face interviews to collect data through questionnaires. The interviews took place at the participants’ doorsteps and lasted approximately 20–25 min.

### 2.2 Measuring

#### 2.2.1 Demographic information form

Demographic information included age, gender, marital status, occupation, education level, and monthly income status.

### 2.3 Health literacy scale

To collect data, the researchers utilized the validated Health Literacy for Iranian Adults (HELIA) questionnaire (22). The scale included 47 items and five subscales as follows: reading health information (4 items; *α =* 0.72); understanding health information (7 items; *α =* 0.86) using a five-point Likert scale ranging from completely difficult (1) to completely easy (5); appraisal of health information (4 items; *α =* 0.77); ability to access health information (6 items; *α =* 0.86); and decision making (12 items; *α =* 0.89) based on five-point Likert scale (always = 5, most of the time = 4, sometimes = 3, seldom = 2 and never = 1).

### 2.4 Morisky medication adherence scale (MMAS-8)

The Persian version of the MMAS-8 scale was used to measure treatment adherence (23). For each item, the response categories were yes/no, with a dichotomous response and a five-point Likert response for the final item. The total score ranges from 0 to 8. Scores of less than 6 indicate low adherence, scores of 6 to <8 indicate moderate adherence, and score = 8 indicates high adherence.

### 2.5 Illness perception scale

To measure illness perception, the Brief Illness Perception Questionnaire (BIPQ-R) was employed (24). This measurement tool includes eight items that are rated on a 0–10 scale. The following five items assess cognitive illness representations: consequences (degree of impact on life), timeline (perceived timeline of illness), personal control (perceived control of illness), treatment control (perceived benefit from treatment), and identity (grade of symptoms associated with the illness). Concern (level of concern about the disease) and emotional affect (level of affect) are two questions that assess emotional illness representations. The last item assesses the comprehensibility/coherence of illness.

### 2.6 Data analysis

Depending on the data distribution type, percentages and frequencies were applied for categorical variables, while mean, standard deviation, median, and quartile deviation were used for continuous variables. The independent samples t-test and one-way ANOVA were used for bivariate comparisons of quantitative variables. The link between HL dimensions and illness perception with treatment adherence was measured using the Pearson correlation.

Three different blocks of independent variables were used to do a hierarchical regression analysis on treatment adherence. Demographic variables made up Block 1. Block 2 came with demographic characteristics and illness perception. The scores from HL dimensions, illness perception, and demographic variables of block three made up this block. To determine the percentage of variance characterized by treatment adherence, we evaluated the adjusted R2 change after inserting each block. The assumptions behind regressions were confirmed by tests for multi-collinearity, normalcy, and significant data points. The threshold for significance was fixed at = 0.05, and the significance level was set to *α =* 0.05. All analyses were conducted using the SPSS 21 software.

## 3 Results

A total of 300 individuals agreed to participate in the study. The median of systolic hypertension was found to be 148.97 mmHg (SD = ±21.20), while the median diastolic hypertension was measured at 103.10 mmHg (SD = ±21.98). Among the participants, 43.3% were male, and most of them were illiterate or had primary education (43.0%). Based on the findings, there was a statistically significant association between age groups, education level, and monthly income status with adherence to therapeutic regimens. Table 1 shows the demographic characteristics and their relationship to adherence to therapeutic regimens.

**Table 1:** The relationship between demographical variables with adherence to medication (*n* = 300).

Variables		*N* (%)	Me ± SD	*p*-value
Age (years)*	60–64	143 (47.7)	5.33 ± 1.56	0.041#
	65 years and older	157 (52.3)	5.68 ± 1.33	
Gender*	Male	130 (43.3)	5.55 ± 1.40	0.700
	Female	170 (56.7)	5.48 ± 1.50	
Level of education**	Primary and illiterate	129 (43.0)	5.48 ± 1.67	0.009#
	Medium	92 (30.2)	5.17 ± 1.42	
	High school and diploma	79 (26.3)	5.78 ± 1.28	
Occupation**	Employee	67 (22.3)	5.47 ± 1.48	0.881
	Housekeeper	131 (43.7)	5.56 ± 1.37	
	Retired	102 (34.0)	5.48 ± 1.55	
Marriage status*	Married	284 (94.7)	5.54 ± 1.45	0.102
	Single	16 (5.3)	4.93 ± 1.48	
Income (month)*	Less than 150 dollars	114 (38.0)	5.78 ± 1.62	0.022#
	150 to 200 dollars	83 (27.7)	5.59 ± 1.23	
	More than 200 dollars	103 (34.3)	5.21 ± 1.50	

*Independent sample *T*-test.

**One way ANOVA test.

#*p*-value < 0.05.

The bivariate associations for HL dimensions and illness perception with adherence to therapeutic regimens are displayed in Table 2. Using the Pearson correlation coefficient test, we observed that adherence to therapeutic regimens had statistically significant relationships with illness perception and all HL dimensions (*p*-value <0.05).

**Table 2:** Bivariate correlation matrix of the relationship between HL dimension, illness perception, and adherence to medication.

Variables	1	2	3	4	5	6	7	Me ± SD
1 = Reading health information	1							12.31 ± 7.92
2 = Ability to access health information	0.575*	1						18.48 ± 8.48
3 = Understanding health information	0.678*	0.844*	1					22.78 ± 8.22
4 = Appraisal of health information	0.478*	0.712*	0.777*	1				13.50 ± 3.86
5 = Decision-making	0.132*	0.407*	0.403*	0.546*	1			33.09 ± 8.69
6 = Illness perception	0.162*	0.272*	0.346*	0.328*	0.422*			49.84 ± 12.99
7 = Treatment adherence	0.278*	0.245*	0.328*	0.201*	0.219*	0.335*	1	5.51 ± 1.45

*Correlation is significant at the 0.05 level (two-tailed).

In Table 3, hierarchical regression was used to assess the predictive validity of demographic variables, illness perception, and HL dimensions over adherence to therapeutic regimens. The results revealed that, in step 1, demographic variables were collectively able to explain 3.2% of the variance in adherence to therapeutic regimens (*p*-value = 0.143). Inclusion of illness perception at step 2 significantly increased 9.6% of the variance explained in hypertension (*p*-value <0.001). In the final step, reading health information, ability to access health information, understanding health information, appraisal of health information, and decision-making were added, which explained an additional 8.7% of the variance (*p*-value <0.001). In total, demographic variables, illness perception, and HL dimensions explained 21.5% of the variance related to adherence to therapeutic regimens.

**Table 3:** Hierarchical linear regression for adherence to medication through demographic characteristics, HL dimensions, and illness perception.

Variables	*ß*	*R*^2^ change	*F* change	SE	*P*-value*
Step 1					
Age	0.019	0.032	1.61	0.011	0.143
Gender	0.084			0.194	
Education level	0.064			0.123	
Job	0.005			0.119	
Marriage	0.095			0.376	
Income	0.119			0121	
Step 2					
Age	0.027	0.096	32.01	0.011	< 0.001
Gender	0.058			0.185	
Education level	0.053			0.117	
Job	0.028			0.113	
Marriage	0.080			0.358	
Income	0.028			0.118	
Illness perception	0.321*			0.006	
Step 3					
Age	0.001	0.87	6.37	0.010	< 0.001
Gender	0.007			0.185	
Education level	0.121			0.117	
Job	0.031			0.109	
Marriage	0.101			0.350	
Income	0.010			0.116	
Illness perception	0.230*			0.007	
Reading health information	0.148*			0.014	
Ability to access health information	0.034			0.018	
Understanding health information	0.348*			0.022	
Appraisal of health information	0.237*			0.035	
Decision-making	0.079			0.012	
Total *R*^2^	–	0.215	–	–	–
Adjusted *R*^2^	–	0.182	–	–	–

*P-value < 0.05.

## 4 Discussion

Adherence to medication is an important factor in controlling and reducing the side effects of diseases, particularly in the older population. Medication adherence varies in older adults due to the effects of various factors. In this study, we examined the determinants of medication adherence among older adults with hypertension.

Based on the findings, there was a statistically significant association between age groups, education level, and monthly income status with adherence to medication. Better medication adherence is affected by aging, high incomes, and high education levels. Previous studies showed that drug adherence was better in patients aged 65–80 years compared to younger hypertensive patients, and age was a significant factor (2, 25, 26). The results revealed that counties with low income were associated with poor medication adherence (27–29). In Iran, health costs increased in the past years and on the other hand, limited resources are concerns in the health system (30). A Scoping Review in Iran demonstrated health costs and amount of out-of-pocket payment are high (31).

Also, employment in informal and formal occupations was considered a significant predictor of medication adherence among people with hypertension (32). In several other studies, educational level was a determinant of medication adherence in hypertensive individuals (1, 25, 33). The main reason for the higher medication adherence among people with higher education can be attributed to their adequate knowledge. It has been reported that both medication adherence and blood pressure control rates were significantly related to hypertension knowledge (34). Medication literacy is an influencing factor for blood pressure control among patients with hypertension (35). These subgroups of demographical variables should be prioritized for interventional programs by health care providers in these patients.

The results of this study demonstrated that adherence to medication had a significant relationship with illness perception and all HL dimensions. This result is in line with previous studies which reported the strong association between medication adherence and health literacy among older adults hypertensive patients (36) and people with hypertension (25, 37). Baharvand et al. (38) found that perceptions of illness consequences could significantly predict treatment adherence. Accordance their results, treatment adherence of hypertensive patients in western Iran is at low level, but their perception of hypertension is at moderate level. A systematic review by Hyvert et al. (29) confirmed an unclear relationship between health literacy and medication adherence, in which 14 studies reported a positive relationship. Lower HL in patients with hypertension made them unable to read or understand the instructions of the prescription drugs; they also failed to communicate effectively with doctors. In addition, hypertensive patients with better HL had better control of their diseases (39). The low level of health literacy caused a 10-year increased risk of cardiovascular disease (40). The higher HL is usually related to better blood pressure control, better hypertension knowledge, better medication adherence, and higher levels of health-related quality of life of the patients; also, health literacy levels affected the outcomes of hypertension health (41).

The results indicated that demographic variables were collectively able to explain 3.2% of the variance in adherence to medication adherence, but this relationship was not significant. The inclusion of illness perception at step 2 significantly increased 9.6% of the variance explained in hypertension. In the final step, reading health information, ability to access health information, understanding health information, appraisal of health information, and decision-making explained an additional 8.7% of the variance. In total, demographic variables, illness perception, and HL dimensions explained 21.5% of the variance in adherence to medication. This finding reinforced the previous report that the complexity of the regimen, health literacy, patient-doctor relationship, family support, and duration of antihypertensive drug use explained 36.1% of the variance in adherence to medication (42). A previous study highlighted that self-efficacy, health belief, patient-provider communication, and medication regimen complexity significantly determined 39.9% of the variation in medication adherence (43). Also, the results of another study revealed that age, sex, education level, anxiety, depression, sleep disturbances, and health literacy accounted for 13.6% of the variance in medication adherence (17). Hence, it is essential to address these predictors in designing patient-centered programs to improve antihypertensive medication adherence in these patients. The current research highlights the need for more specific interventions aiming at promoting HL in hypertension health outcomes and its determinants.

This study had some limitations. First, the participants in this research were selected from a limited area in Iran; therefore, the result cannot be generalized. Second, the measurement of medication adherence was based on self-reported questionnaires, which may introduce recall bias. Third, the sample size was small; studies with larger samples are suggested to be conducted in the future. Fourth, we used Morisky questionnaire. However, after the implementation of the study (between February and May 2023), some concerns have been raised regarding the questionnaire’s statistical analysis in September 2023. Therefore, future studies should use this questionnaire more cautiously.

## 5 Conclusion

According to the results, adherence to medication had a significant relationship with illness perception and all HL dimensions. Overall, demographic variables, illness perception, and HL dimensions explained 21.5% of the variance related to medication adherence. Policymakers, pharmacists and health educators should develop interventions to enhance medication adherence by improving illness perception and HL dimensions among patients. It is also important to address demographic variables in designing patient-centered programs.

## Data availability statement

The raw data supporting the conclusions of this article will be made available by the authors, without undue reservation.

## Ethics statement

The studies involving humans were approved by the Faculty of Ethics Committee in Sarab Medical Sciences. The studies were conducted in accordance with the local legislation and institutional requirements. The participants provided their written informed consent to participate in this study.

## Author contributions

TB: Conceptualization, Formal analysis, Investigation, Methodology, Project administration, Supervision, Validation, Writing – original draft, Writing – review & editing, Resources. SR: Writing – original draft, Writing – review & editing. SP: Data curation, Writing – original draft, Writing – review & editing. AL: Writing – original draft, Writing – review & editing. KM: Conceptualization, Formal analysis, Investigation, Methodology, Project administration, Supervision, Validation, Writing – original draft, Writing – review & editing.
